# Tracheal Stenting with Dumon Stent – A Novel Treatment Modality for Tracheal Stenosis

**DOI:** 10.1055/a-2858-1040

**Published:** 2026-09-11

**Authors:** Acharya Anand, Phani Bhushan I., Kondra Swamy, Sampath Rao Dudapaka, V Nair Lakshmi, Jambula Mohana, P Ravali, Ullas Nair Vrinda

**Affiliations:** 1 Assistant Professor of Department of ENT, Government ENT Hospital, Koti, Telangana, India

**Keywords:** Tracheal Stenosis, Tracheostomy, Iatrogenic, Stents (Dumon's Airway stent)

## Abstract

**Introduction:**

Tracheal stenosis is defined as pathological narrowing of the trachea. Tracheal stenosis can be caused by various factors but endotracheal intubation and tracheostomy are the most common causes of acquired benign tracheal stenosis. Airway narrowing due to inflammation or scar tissue impairs normal breathing and may predispose to upper and lower respiratory infections. The Goal of treatment for tracheal stenosis is to restore a patent airway and glottic competence.

**Objective:**

To determine the efficacy and complication profile of Dumon's stent in treatment for tracheal stenosis.

**Methods:**

An observational prospective cohort study was conducted at a tertiary care center from January 2018 to December 2023. All patients presenting with tracheal stenosis resulting from post-intubation, post-tracheostomy, or corrosive acid ingestion were considered. A complete history was obtained followed by physical examination and necessary investigations.

**Results:**

Mean age of study subjects was 22.5 years and male preponderance (60.7%) was noted. Most common cause of tracheal stenosis was found to be endotracheal intubation (57.1%). Dumon stent placement demonstrated no complications such as granulation tissue formation, scarring or restenosis.

**Conclusion:**

Post-intubation airway stenosis is the most common cause of tracheal stenosis. Surgical management is the definitive treatment for tracheal stenosis. In this study, Dumon's stent showed no recurrences. These findings suggest that Dumon stent placement is a safe and viable alternative to laryngotracheal end-to-end anastomosis.

## Introduction

Tracheal stenosis is most often a benign acquired iatrogenic condition characterized by pathological narrowing of the trachea that restricts normal breathing. Tracheal stenosis is a potentially life-threatening consequence following prolonged intubation or tracheostomy. 1 The incidence of post-intubation tracheal stenosis ranges from 0.6–21%. 2 3 Improvement in tracheostomy techniques, such as percutaneous dilatation, has reduced the incidence of symptomatic tracheostomy-related stenosis to 0.6–6%. 4 5 Recent literature suggests that some degree of stenosis develops in up to 20–30% of patients with tracheostomy, and 1–7% of patients develop symptomatic tracheal stenosis, including stridor, cough and shortness of breath. 6 7 An average intubation duration of 2 weeks, or tracheostomy with cuffed tubes for 4 weeks, increases the risk of airway stenosis. Patients typically present with stenosis 4–5 weeks after extubation and approximately 4 weeks after tracheostomy decannulation. 8

Benign acquired tracheal stenosis may rarely result from corrosive acid ingestion, which can cause mucosal erosion of the larynx and trachea if the acid is aspirated. Caustic ingestion causes thrombosis of small vessels, leading to inflammation, granulation tissue formation, fibrosis, and stricture formation in the airway. 9

Other rare causes of tracheal stenosis include irradiation, inhalational burns, tuberculosis, bacterial tracheitis, histoplasmosis, diphtheria, and collagen vascular diseases such as Wegener's granulomatosis, relapsing polychondritis, polyarteritis, and scleroderma. 10

## Pathophysiology and Molecular Mechanisms 11

Tracheal stenosis involves complex inflammatory–fibrotic cascades that progress through several pathophysiological stages. After mucosal injury from intubation or tracheostomy, prolonged ischemia and mechanical trauma trigger inflammatory responses characterized by the recruitment of macrophages and T cells, leading to increased production of pro-inflammatory cytokines such as interleukin-6 (IL-6), tumor necrosis factor-alpha (TNF-α), and transforming growth factor-beta (TGF-β). TGF-β plays a central role in activating fibroblasts and myofibroblasts, which excessively produce extracellular matrix components, particularly collagen types I and III. This pathological fibrosis leads to progressive narrowing of the airway lumen and, if untreated, culminates in symptomatic stenosis. Understanding these molecular mechanisms is crucial for selecting appropriate treatment modalities that either interrupt the fibrotic cascade or manage established stenosis.

Tracheal stenosis can be classified as simple or complex stenoses. Simple stenoses are lesions with endoluminal occlusion of a short segment (<1 cm), absence of tracheomalacia, and no loss of cartilaginous support. Complex stenoses are lesions with extensive scarring (>1 cm), varying degrees of cartilage involvement, circumferential contraction scarring, or stenosis associated with malacia and inflammation. 12

There is no fixed approach to laryngotracheal stenosis; the treatment modality is determined based on airway anatomy, site of involvement, length and luminal narrowing of the stenotic segment, and laryngeal and tracheal function, patient-related factors, and available facilities. The goal of treatment is to reconstruct the airway while preserving phonation and glottic competency to prevent cough and aspiration.

Bronchial endoscopy is required to confirm the diagnosis of tracheal stenosis and determine stenosis type. Treatment options for tracheal stenosis include bronchoscopic balloon dilatation, laser cauterization of scarring, radiofrequency coblation, stent placement, and Montgomery tube placement. However, the most effective curative treatment for tracheal stenosis remains the surgical approach—tracheal resection and end-to-end anastomosis (TRE). 12

The surgical approach has a success rate of 71–95%, with an operative failure rate of 3.7% and a mortality rate of 2.4%. Although surgery is considered one of the ideal treatment options for tracheal stenosis, it cannot be offered as first-line treatment to all patients. Patients with medical comorbidities such as diabetes mellitus, obesity, poor general health, and other pre-existing conditions have been shown to have increased risk of postoperative complications. Complications following resection and anastomosis include granulation tissue formation, tracheal restenosis, and fistulae to surrounding structures, such as the innominate artery (leading to Tracheoinnominate fistula [TIF]) or esophagus (leading to Tracheoesophageal fistula [TEF]). Other post-surgical complications include laryngeal edema, glottic dysfunction, and rarely, recurrent laryngeal nerve injury. 13 14

Given the technical demands of airway endoscopy and stent placement, procedural experience significantly influences outcomes. The procedural learning curve in tracheal stenosis management represents an important consideration when evaluating treatment efficacy and safety across different centers and practitioners. Appropriate training using ex vivo and animal models may enhance procedural competency and reduce associated complications. 15

Bronchoscopic methods, including dilatation, laser-assisted dilatation, cryotherapy, and stent placement, can be applied individually or in combination in subjects for whom surgical approach is contraindicated or carries greater risk of complications. 14 16 Micro-debrider and coblation are considered safe and cost-effective alternatives for clearing airway stenosis when stenosis or scarring is in proliferative stages. Laser resection is also an effective alternative but carries greater risk of damaging underlying cartilage. 10 17 Dumon airway stents are silicone prostheses with regularly placed external studs with textured ends to prevent migration or rotation. These stents have demonstrated effective results in treating acquired tracheal stenosis and have shown lower rates of complications, such as fibrotic scarring or bottleneck stricture, which are commonly reported after balloon bronchoplasty, laser resection, and end-to-end anastomosis. 18

## Objectives

The present study was conducted to: (1) evaluate various treatment modalities and their outcomes for tracheal stenosis, and (2) determine the efficacy and complication profile of Dumon stent use, specifically in the management of complex tracheal stenosis.

## Methods

### Study Design and Setting

An observational prospective longitudinal cohort study was conducted in the Department of Otorhinolaryngology and Head & Neck Surgery at a tertiary care center in Hyderabad, India, from January 2018 to December 2023. The study design tracks patient outcomes prospectively from treatment initiation through follow-up. Institutional ethics committee clearance was obtained prior to commencement of the study. All patients presenting with tracheal stenosis following intubation, tracheostomy, or corrosive acid poisoning were considered for the study after obtaining informed consent. Subjects with tracheal stenosis due to malignancies were excluded from the study.

### Inclusion and Exclusion Criteria (PICO/PICOTS Framework)

Population: Adult patients diagnosed with benign acquired tracheal stenosis

Intervention: Various endoscopic and surgical treatment modalities (coblation excision, laser cauterization, balloon dilatation, Montgomery tube placement, Dumon stent placement, or tracheal resection and end-to-end anastomosis)

Comparison: Comparative analysis of outcomes across different treatment modalities

Outcomes: Primary outcomes—recurrence and complications; Secondary outcomes—treatment efficacy, stenosis resolution, symptom improvement

Study Design: Observational prospective cohort study

Time Frame: January 2018–December 2023, with follow-up duration varying by treatment group

### Data Collection and Clinical Assessment

A complete history was obtained, including age, sex, underlying conditions, duration of endotracheal intubation and/or tracheostomy, time interval between extubation and occurrence of first symptoms, and presenting symptoms. Video laryngoscopy and lateral neck radiography were performed for all patients, followed by axial, coronal and sagittal CT imaging of the neck. Diagnostic bronchoscopy was performed for all patients, and stenoses were classified based on bronchoscopic findings. Web-like or simple stenoses were defined as short-segment (<1 cm) membranous concentric stenoses without cartilage damage, according to the McCaffrey classification system (Grade I: <1 cm subglottic or tracheal stenosis; Grade II: >1 cm subglottic stenosis; Grade III: >1 cm subglottic and tracheal stenosis; Grade IV: any glottic involvement). All other stenoses, including those with extensive scar tissue (>1 cm), circumferential hourglass-like contraction scarring, or malacia, were classified as complex stenoses.

Definition of Recurrence: Tracheal stenosis was considered cured when patients remained symptoms-free, including during exertion, for 1 year following the last therapeutic intervention. Recurrence was operationally defined as either symptomatic restenosis (return of dyspnea, stridor, or voice change) or bronchoscopic evidence of restenosis (luminal narrowing ≥50% of baseline diameter) requiring re-intervention.

Assessment of Complications: Complications were assessed using both clinical evaluation and scheduled bronchoscopic surveillance. For patients treated with stent placement, routine bronchoscopy was performed at 1 month and then every 6 months to assess for stent position, migration, granulation formation, mucus retention, and restenosis. For patients treated with coblation, laser cauterization, or balloon dilatation, physical examination and bronchoscopy were performed at 3-week intervals during the first 3 months and then every 6 months thereafter. Complications documented included granulation tissue formation, mucostasis, stent migration, restenosis, and scarring.

Preoperative routine investigations, viral screening, coagulation parameters, and pre-anesthetic evaluation were performed to assess fitness for surgery, and written informed consent was obtained from all patients after explanation of procedure risks and benefits. Median follow-up duration was 12 months (range: 6–36 months) for the Dumon stent subgroup (n = 7) and 12 months (range: 6–24 months) for other modalities (n = 21), reflecting variable enrollment over the 6-year study period.

### Treatment Allocation and Procedural Methodology

Endoscopic examination of the larynx and trachea under general anesthesia was performed, and the degree and length of stenosis was graded using the McCaffrey classification system. Treatment modality was determined based on stenosis grade and type (simple vs. complex), history of previous treatment failures, patient comorbidities, and clinical factors – not by randomization. This treatment allocation strategy introduced potential selection bias and confounding by indication, particularly because Dumon stents were preferentially used for complex stenoses and recurrent simple stenosis, whereas other modalities were primarily used for initial simple stenosis management. This distinction is acknowledged in the statistical analysis and discussion.

**Fig. 1:**
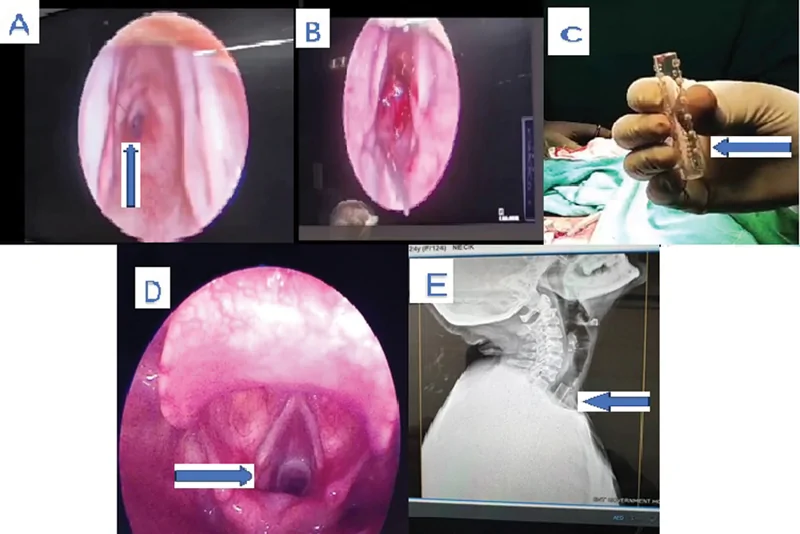
(A) Stenotic Segment; (B) After Bronchoscopic Dilatation; (C) Dumon stent with studs; (D) After placing Stent in Trachea; (E) Radiograph Neck Lateral View at 3-months follow-up showing stent in situ.

For simple stenoses, scar tissue and webbing removal was performed using either microdebrider or coblation, followed by rigid bronchoscope dilatation without stenting. Complex stenoses (as shown in Fig. 1A ) were managed under general anesthesia administered through the tracheostomy tube. The skin around the tracheostomy opening was excised to create a raw area, preventing ingrowth of skin and suprastomal fibrous tissue narrowing. The area was excised and dilated, confirmed by direct laryngoscopy ( Fig. 1B ). The Dumon stent ( Fig. 1C ) was then introduced through the tracheal stoma and positioned ( Fig. 1D ) such that its upper and lower openings bridged across the tracheostomy wound. Any tracheal defect from long-standing tracheostomy was supplemented with cartilage grafting harvested from the auricular cartilage.

Post-stent placement follow-up was conducted at 1 month and subsequently every 6 months. Physical examination and lateral neck radiography were performed at follow-up visits to assess stent position and detect stent migration ( Fig. 1E ). Dumon stent placement was also considered the treatment of choice in patients with simple stenosis who experienced treatment failure on three consecutive occasions.

Patients treated for simple stenosis through scar tissue and webbing removal with either microdebrider or coblation followed by rigid bronchoscope dilatation were assessed via physical examination and bronchoscopy at 3-week intervals during the first 3 months and once every 6 months thereafter.

## Results

### Patient Demographics and Clinical Characteristics

A total of 28 patients with tracheal stenosis were included during the study period. Simple stenosis was identified in 20 cases (71.4%), and complex stenosis in 8 cases (28.6%). The majority of patients (32.2%) were in the 21–30 years age group, with a mean age of 22.5 years. Male predominance was noted (60.7%), with a male-to-female ratio of approximately 1.5:1. Endotracheal intubation (57.1%) and tracheostomy (21.4%) were the most common etiological factors ( Table 1 ). Voice change (96.4%), shortness of breath on exertion (57.1%), and stridor (42.8%) were the most common presenting symptoms.

**Table 1:** Demographic and clinical profile of study participants

Age (years)	Frequency (%)
0–10	6 (21.4)
11–20	6 (21.4)
21–30	9 (32.2)
31–40	5 (17.8)
41–50	1 (3.6)
51–60	1 (3.6)
**Sex**	**Frequency (%)**
Male	17 (60.7)
Female	11 (39.3)
**Aetiology**	**Frequency (%)**
Endotracheal intubation	16 (57.1)
Tracheostomy	6 (21.4)
Corrosive acid poisoning	1 (3.6)
Congenital	2 (7.2)
Post traumatic	3 (10.7)
**Clinical features (Multiple features were present)**	**Frequency (%)**
Shortness of breath on exertion	16 (57.1)
Stridor	12 (42.8)
Change of voice	27 (96.4)

The mean duration from extubation to stenosis development was 31.1 days (range: 14–60 days). For tracheostomy-related stenosis, the mean time from decannulation to symptom onset was 20.2 days (range: 7–45 days). Immediate onset stenosis (mean: 1 day) was observed in patients with a history of corrosive acid ingestion ( Table 2 ).

**Table 2:** Duration of Aetiological cause and symptomatology among study participants

Aetiology	Mean number of days of intubation/tracheostomy done (Range)	Mean interval of duration of symptomatic stenosis development after extubation/decannulation (Range)
Endotracheal intubation	15.5 (11–20)	31.1 (11–55)
Tracheostomy	28.5 (26 -31)	20.2 (9–31)
Corrosive acid poisoning		1

### Treatment Allocation and Outcomes

Based on diagnostic bronchoscopy findings and treatment allocation criteria described in the methodology, simple stenoses were managed using coblation excision, laser cauterization, balloon dilatation, and Montgomery tube placement ( Table 3 ). Among complex stenoses (n = 8), 7 cases (87.5%) were managed with Dumon stent placement, while 1 case underwent tracheal resection and end-to-end anastomosis ( Table 3 ).

**Table 3:** Treatment modalities offered for different types of stenoses

Type of stenosis (%)	Coblation Excision (%)	Laser (%)	Montgomery tube (%)	Balloon dilatation (%)	Dumon Stent (%)	End to end anastomosis (%)
Simple stenosis (n = 20; 71.4%)	8 (40%)	1 (5%)	5 (25%)	6 (30%)	–	–
Complex stenosis (n = 8; 28.6%)	–	–	–	–	7 (87.5%)	1 (12.5%)
Total (n = 28; 100%)	8 (28.6%)	1 (3.6%)	5 (17.8%)	6 (21.4%)	7 (25%)	1 (3.6%)

### Complication Profile

Among patients treated with coblation excision, granulation tissue was the most common complication, observed in 100% of cases, with concurrent scarring and restenosis noted in all patients. For Montgomery tube placement, granulations were documented in 80% of cases, mucostasis in 100% of cases, and restenosis in 60% of cases. Among patients undergoing balloon dilatation, restenosis was observed in 50% of cases ( Table 4 ).

**Table 4:** Distribution of complications reported on follow – up visits

Treatment modality	Granulations (%)	Scarring & Restenosis (%)	Mucostasis (%)	Stent Migration (%)	Restenosis (%)
**Coblation excision (n** **=** **8)**	8 (100)	4 (50)	–	–	6 (75)
**Laser (n** **=** **1)**	–	–	–	–	–
**Montgomery tube (n = 5)**	4 (80)		5 (100)		3 (60)
**Balloon dilatation (n = 6)**	–	–	–	–	3 (50)
**Dumon Stent (n = 7)**	–	–	1 (14.2)	–	–
**End to end anastomosis (n = 1)**	–	–	–	–	–

Notably, in the Dumon stent group (n = 7), no granulations, scarring, or stent migration were observed. Only one patient developed mucostasis, which responded effectively to conservative medical management ( Table 4 ). The patient treated with tracheal resection and end-to-end anastomosis experienced no complications during the follow-up period.

### Statistical Comparison

Statistical analyses was performed to compare the efficacy of Dumon stent placement with other treatment modalities in terms of complications and recurrence. Complications were significantly less frequent in the Dumon stent group than in other treatment modalities (p < 0.05; Yates' correction applied). Similarly, recurrence rates were statistically significantly lower in patients treated with Dumon stent placement (p < 0.05; Yates' correction applied) ( Table 5 ). However, given the small sample size of the Dumon group (n = 7) compared to simple stenosis groups (n = 20), and the inherent selection bias resulting from preferential allocation of complex cases to Dumon stenting, these comparisons should be interpreted with caution. Direct statistical comparison between groups with different clinical presentations and disease severity may not fully capture treatment efficacy across stenosis types.

**Table 5:** Comparison of complications and recurrences among Dumon stent vs various treatment modalities

	All other treatments (%)	Dumon stent (%)	Total (%)	Yates corrected Chi square value; p value
**Complications present (%)**	18 (94.7)	1 (5.3)	19 (100)	9.2; p = 0.002*
**Complications absent (%)**	3 (33.3)	6 (66.7)	9 (100)	
**Total (%)**	21 (75)	7 (25)	28 (100)	
**Recurrence present (%)**	12 (100)	0 (0)	12 (100)	4.8; p = 0.02*
**Recurrence absent (%)**	9 (56.2)	7 (43.8)	16 (100)	
**Total (%)**	21 (75)	7 (25)	28 (100)	

*p value <0.05; statistically significant.

## Discussion

Tracheal stenosis represents both congenital and acquired narrowing of the trachea, resulting from pathological scar formation and leading to exertional dyspnea, phonation difficulties, and stridor. This study evaluated various treatment modalities and their associated outcomes in managing both simple and complex tracheal stenosis.

### Patient Demographics and Etiology

In this study, the majority of cases (32.2%) occurred in patients aged 21–30 years, with male predominance (60.7%), findings consistent with previous reports. 19 20 The most common etiological factor was post-intubation stenosis (57.1%), followed by post-tracheostomy stenosis (21.4%). This finding is consistent with the known pathophysiology: mechanical trauma to the tracheal cartilage causes mucosal edema and ischemia, leading to subsequent stenosis formation. These findings align with reports by Padmanabhan et al., Rea et al., and Parida et al. 19 20 21

### Complications Following Endoscopic Management of Simple Stenosis

In this cohort, the most common complications following treatment of simple stenoses included granulation tissue formation, restenosis, and mucostasis. All patients (100%) treated with coblation excision developed granulation tissue, with concurrent scarring and restenosis. Patients treated with Montgomery tube placement experienced mucostasis (100%), granulations (80%), and restenosis (60%). Restenosis was documented in 50% of patients following balloon dilatation ( Table 4 ). These findings are consistent with prior studies: Padmanabhan et al. 19 reported granulation tissue in 22%, restenosis in 11.1%, and stent migration in 5.6%, while Nikolovski et al. 22 reported granulation tissue in 27%, restenosis in 19%, and stent migration in 11%.

### Outcomes with Complex Stenosis Management

For complex stenoses, Dumon stent placement and tracheal resection with end-to-end anastomosis were utilized. The single patient treated with tracheal resection and end-to-end anastomosis experienced no complications. In the Dumon stent group (n = 7), no granulations, restenosis, or stent migration were observed. One patient (14.3%) developed mucostasis, which was successfully managed conservatively ( Table 4 ). These findings suggest favorable short- to medium-term outcomes, though long-term durability requires continued surveillance. These results are consistent with prior reports demonstrating the efficacy of silicone stents: Tsakiridis et al. reported excellent results with minimal granulations and no restenosis with silicone stents, 23 and Martinez-Ballarin et al. documented migration in 17.5% of cases, granulations in 6.3%, and mucostasis in 6.3%. 24

However, it is important to acknowledge that the favorable complication profile observed with Dumon stents in this study may reflect case selection bias, as Dumon stents were primarily allocated to complex stenoses with greater disease severity. Comparative studies by Chen et al., Lin et al., and Guo et al. report variable complication rates: Chen et al. documented a 40.74% curative rate and 75.49% efficacy with Dumon stents, with 25.04% stent migration, 15.66% granulations, and 23.82% mucus retention. 25 Lin et al. reported that silicone stents were beneficial except for stent migration complications, 26 and Guo et al. documented 28.57% granulations, 14.29% stent migration, and 21.43% requiring stent removal. 27 The variability in reported outcomes across studies may reflect differences in operator experience, follow-up duration, and patient populations, underscoring the importance of procedural learning curves in complex airway management.

### Study Limitations and Future Directions

This study has several important limitations. First, the small sample size, particularly for the Dumon stent subgroup (n = 7), limits the generalizability and precision of findings. Second, the non-randomized treatment allocation introduces selection bias and confounding by indication, making direct statistical comparisons between groups challenging. Third, the study was conducted at a single tertiary center, limiting external validity. A multicentric prospective study with larger sample sizes, standardized follow-up protocols, and stratification by stenosis grade and type would be needed to determine the accuracy and precision of Dumon stent efficacy in treating tracheal stenosis and to fairly compare treatment modalities across disease severity levels.

## Conclusion

Dumon stents are durable silicone prostheses resistant to microbial contamination and are associated with minimal granulation tissue formation. In this study, Dumon stent placement in patients with complex tracheal stenosis was found to be safe and effective, with a favorable complication profile. Dumon stent placement may be considered a valuable treatment option for complex stenoses and recurrent simple stenoses in appropriately selected patients, demonstrating favorable short- to medium-term outcomes.
